# Feasibility of decentralized cervical cancer survivorship care at district hospitals in Rwanda: a multi-site pilot implementation study

**DOI:** 10.1093/oncolo/oyag275

**Published:** 2026-07-16

**Authors:** Fidel Rubagumya, Vincent Kwizera, Isabelle Mutetiwabo, Samson Habimana, Francois Sebashi, Febronie Muhorakeye, Nina S N Ngowi, Ilir Hoxha, Laetitia Nyirazinyoye, Leon Mutesa

**Affiliations:** Department of Internal Medicine, University of Rwanda, Kigali, Rwanda; Department of Oncology, Rwanda Military Teaching Hospital, Kigali, Rwanda; Dartmouth Cancer Center, Geisel School of Medicine, Hannover, NH, United States; Department of Research, The Rubagumya Lab—Rwanda, Kigali, Rwanda; Department of Research, The Rubagumya Lab—Rwanda, Kigali, Rwanda; Department of Research, The Rubagumya Lab—Rwanda, Kigali, Rwanda; Department of Obstretrics and Gynecology, Nyagatare District Hospital, Nyagatare, Rwanda; Department of Obstretrics and Gynecology, Ruhengeri L2 Hospital, Musanze, Rwanda; Department of Obstretrics and Gynecology, Kirehe District Hospital, Kirehe, Rwanda; Department of One Health, University of Global Health Equity, Kigali, Rwanda; Department of Research, Evidence Synthesis Group, Kosovo, Rwanda; School of Public Health, University of Rwanda, Kigali, Rwanda; Center for Human Genetics, College of Medicine and Health Sciences, University of Rwanda, Kigali, Rwanda

**Keywords:** cervical cancer, survivorship care, district hospitals, Rwanda

## Abstract

**Background:**

Cervical cancer is the leading cause of cancer death among women in sub-Saharan Africa, yet structured survivorship care remains concentrated in tertiary centers and is often inaccessible to survivors living far from oncology services. Decentralizing follow-up to district hospitals may reduce patient burden and improve continuity of care, but prospective implementation evidence from low- and middle-income country settings remains limited. We evaluated the feasibility, acceptability, fidelity, safety, and patient burden of gynecologist-led district-hospital cervical cancer survivorship follow-up in Rwanda.

**Methods:**

This prospective, mixed-methods, multi-site pilot study enrolled 12 cervical cancer survivors at 4 purposively selected district hospitals. Participants underwent quarterly gynecologist-led follow-up over 12 months. Prespecified implementation outcomes, guided by Proctor’s taxonomy, included on-time visit completion within 14 days of the planned date as the primary feasibility metric, with a threshold of ≥80%; clinician and patient acceptability and appropriateness using acceptability of intervention measure (AIM)/ intervention appropriateness measure (IAM); clinician feasibility using feasibility of intervention measure (FIM); and visit-level protocol fidelity assessed by structured chart review. Safety was assessed through clinical record and referral documentation review. Patient travel time and transport costs were compared with self-reported pre-enrollment tertiary-center data. consolidated framework for implementation research-informed interviews explored contextual determinants.

**Results:**

Twelve women were enrolled, with median age 69 years (interquartile range [IQR] 54-72) and FIGO stage II-IVA disease. Of 60 scheduled visits, 51 were completed within the prespecified 14-day window (85.0%; 95% confidence interval [CI] 73.4-92.9), meeting the feasibility threshold. Site-level on-time completion ranged from 66.7% to 93.8%. Acceptability, appropriateness, and feasibility were high among clinicians and patients: clinician AIM 4.5, IAM 4.3, FIM 4.3; patient AIM 4.4 and IAM 4.2, with all respondents scoring ≥4. Visit-level protocol fidelity was low, with median fidelity of 64.2% (IQR 58.9-67.4), and only 4.3% of visits met the ≥85% threshold. No serious adverse events attributable to district-level follow-up were identified under passive surveillance. Median travel time decreased by 75%, and transport costs by 71%.

**Conclusion:**

District-hospital cervical cancer survivorship follow-up was acceptable, feasible, and reduced patient burden, but fidelity strengthening and prospective safety monitoring are needed before scale-up.

Implications for Practice This pilot study suggests that cervical cancer survivorship follow-up can be feasibly decentralized to district hospitals in Rwanda, reducing travel time and transportation costs while maintaining high patient and clinician acceptability. For low-resource settings where oncology services are centralized, gynecologist-led district-hospital follow-up may improve access and continuity of survivorship care. However, low visit-level fidelity highlights that scale-up should include standardized documentation, clear referral pathways, audit and feedback, refresher training, and prospective safety monitoring to ensure quality recurrence surveillance and late-effect management.

## Introduction

Cervical cancer is the leading cause of cancer-related death among women in sub-Saharan Africa, accounting for over 117 000 deaths annually on the continent, more than 90% of which occur in low- and middle-income countries (LMICs).^1^^,^^2^ In Rwanda, cervical cancer represents the largest share of female cancer-related mortality, and most women present with locally advanced disease (FIGO stage II-IVA), reflecting persistent gaps across the cancer care continuum.^3^^,^^4^ Rwanda has made extraordinary progress in cervical cancer prevention with Human Pappilomavirus (HPV) vaccination coverage exceeding 90% among eligible adolescent girls, and the Ministry of Health’s Mission 2027 strategy targets cervical cancer elimination 3 years ahead of WHO global targets through expanded screening, treatment, and referral systems.^5^ Yet screening coverage remains low 18% in 2023 to 30% in 2025 which is far below the 70% WHO target and the growing cohort of women who survive treatment faces a critical and underserved gap of structured, long-term survivorship care.^6^^,^^7^

Structured survivorship care, encompassing surveillance for recurrence, management of treatment-related late effects (including genitourinary toxicity, lymphedema, neuropathy, and psychosexual morbidity), psychosocial support, and health promotion, is now recognized as an essential component of comprehensive cancer control.^8^^,^^9^. Evidence-based frameworks from the American Society of Clinical Oncology (ASCO) and other professional societies provide structured guidance for post-treatment surveillance and late-effects management.^10^ However, these frameworks were designed for high-resource health systems with specialist oncology workforces and are largely inapplicable to LMIC settings where gynecological oncologists are scarce, and survivors far outnumber specialists.^11^^,^^12^ In the absence of adapted LMIC survivorship models, most women who complete cervical cancer treatment in sub-Saharan Africa receive no structured post-treatment follow-up.

In Rwanda, specialized oncology services are concentrated at 2 tertiary centers, Butaro Level II Teaching Hospital and the Rwanda Cancer Center at Rwanda Military Teaching Hospital, serving a geographically dispersed population of more than 14 million. Traveling to these facilities imposes a substantial financial burden that compels many survivors to forego follow-up.^13^^,^^14^ This financial burden directly contributes to delayed detection of recurrence and unmanaged late treatment effects. Rwanda’s district hospitals, each staffed by at least 1 gynecologist and geographically closer to most patients than tertiary centers, with documented improvements in nationwide access to gynecologic care, represent a feasible platform for decentralized survivorship care.^15^^,^^16^ However, no prospective evidence exists on the feasibility, clinical fidelity, acceptability, or safety of structured district-level cervical cancer survivorship follow-up in Rwanda or any comparable LMIC context.

To address this evidence gap, we report a prospective, multi-site, mixed-methods pilot implementation study of decentralized cervical cancer survivorship care at district hospitals in Rwanda.

## Patients and methods

### Study design

We conducted a prospective, mixed-methods, multi-site pilot feasibility and implementation study, guided jointly by Proctor et al.'s taxonomy of implementation outcomes^17^^,^^18^ and the consolidated framework for implementation research (CFIR).^19^^,^^20^ The quantitative component evaluated prespecified implementation benchmarks; the qualitative component used CFIR-informed framework analysis to explain barriers, facilitators, and inter-site variation.

### Setting and participants

Four district hospitals were purposively selected for exploratory and contextual reasons, intended to capture province-level variation in implementation determinants, the study was not powered for inter-site comparison. Historical data on patients’ volume was also considered. Site selection was also guided by the presence of gynecologists and the expectation that participant numbers would be reasonably balanced across the selected hospitals. Reporting was guided by relevant CONSORT pilot and feasibility items adapted for a single-arm design and by GRAMMS principles for mixed methods studies.^21^^,^^22^ At each site, 1 gynecologist underwent a standardized 1-day pre-study training workshop in cervical cancer survivorship care delivery before participant enrollment. The workshop was delivered jointly by the principal investigator (oncology trained), a senior gynecologist, and the study research coordinator, and covered (1) survivorship care plan use, (2) structured pelvic examination, (3) red-flag identification and escalation, and (4) standardized documentation. A short post-training competency check (case-based knowledge questions and supervised pelvic examination) was completed before implementation. Eligible participants were women aged ≥18 years with histologically confirmed cervical cancer; had completed primary concurrent chemoradiotherapy within the preceding 3-12 months; had no clinical or radiological evidence of disease recurrence at enrollment, as determined by combined clinical and radiological review at the Rwanda Cancer Center/Rwanda Military Teaching Hospital using a standardized end-of-treatment assessment template; and had resided within the defined catchment area of the participating district hospital. Women unable to provide written informed consent were excluded. The target enrollment was 12 participants (∼3 per site), consistent with pilot feasibility guidance.^18^ Each enrolled participant was scheduled for quarterly (3-monthly) follow-up visits over 12 months, yielding a maximum of 4 per-protocol visits per participant.

### Intervention

The decentralized survivorship care intervention comprised 4 integrated components:

1. A 1-day capacity-building workshop for district hospital gynecologists and research assistants, covering the survivorship care model, follow-up schedule, red-flag escalation triggers, and documentation requirements.
2. Provision of an adapted ASCO Cervical Cancer Survivorship Care Plan to each participant at the time of transition from tertiary care, serving as a structured handover document communicating treatment history, late-effect surveillance priorities, and referral criteria. ASCO guidance was selected because it provides the most operationally specific, item-level survivorship care plan for cervical cancer. Adaptation comprised (1) condensing surveillance items to those feasible at district level, (2) translating patient-facing content to Kinyarwanda, and (3) adding red-flag triggers consistent with the Rwandan referral pathway. The adapted tool was piloted with 2 gynecologists and 3 survivors outside the study before use; minor wording changes followed.
3. Quarterly telephone check-ins by a trained research assistant between clinic visits to administer standardized quality-of-life assessments (EORTC QLQ-C30 and QLQ-CX24) and document interim symptoms. We note that these telephone check-ins were a research-support component rather than a routine programme element.
4. Quarterly district-hospital visits during which the gynecologist performed a structured history and physical examination (including pelvic examination) using a shared structured visit checklist with predefined documentation fields for pelvic findings, investigations, and referral decisions, documented findings, ordered investigations if clinically indicated, and determined whether referral back to the tertiary center was warranted.

### Outcomes and definitions

Implementation outcomes were defined a priori according to Proctor et al.'s taxonomy.^17^ Prespecified success thresholds were established by the study team as pragmatic progression criteria for this pilot implementation study, informed by the study objectives, local operational context, and the need to identify signals of acceptable performance.^23^^,^^24^ Because standardized interpretive cutoffs are not available for acceptability of intervention measure (AIM), intervention appropriateness measure (IAM), and feasibility of intervention measure (FIM), a mean score of ≥4.0 on the 5-point scale was used to indicate favorable acceptability, appropriateness, and feasibility. Likewise, thresholds of ≥80% for timely follow-up completion and ≥85% for visit-level fidelity were prespecified as benchmarks for satisfactory implementation performance. Feasibility was defined as the proportion of scheduled follow-up visits completed within 14 days of the planned date. Acceptability and appropriateness were measured via the validated AIM and IAM,^25^ while clinician feasibility was assessed using the FIM.^25^

Visit-level fidelity was defined as the proportion of required protocol elements completed per visit assessed via structured chart review. These elements included history and physical (pelvic) examination, documentation of findings, investigations ordered, and referral decisions. We note that this measure reflects documented task completion rather than the clinical quality or appropriateness of the assessment. The prespecified high-fidelity threshold was ≥85% of elements completed per visit. On-time visit completion (≥80% within 14 days) was prespecified as the primary progression criterion; acceptability/appropriateness, visit-level fidelity, and safety were secondary progression criteria. The ≥4.0 cut-point for AIM/IAM/FIM is a study-defined pragmatic benchmark, not a validated cutoff. Baseline implementation fidelity was separately assessed as completion of pre-study structural requirements (training, care plan delivery, referral note transmission). Safety was defined as the absence of serious adverse events attributable to decentralized follow-up and timely escalation (within ≤7 days from red-flag identification to definitive action). Serious adverse events of interest were (1) confirmed disease recurrence detected outside the protocol window, (2) treatment-related toxicity requiring hospitalization, and (3) delays ≥7 days from red-flag identification to definitive action. Attribution to district-level follow-up was independently adjudicated by 2 clinicians (F.R. and V.K.), with discrepancies resolved by consensus. Missed recurrences and delayed escalations were tabulated separately. Units of analysis were: per visit (visit completion, visit-level fidelity), per respondent (AIM/IAM/FIM), and per participant (safety, referral). Patient-level burden was measured by comparing self-reported round-trip travel time, out-of-pocket transportation costs (Rwandan francs, RWF), and workdays lost per district-hospital visit with retrospectively self-reported values anchored to each participant’s most recent pre-enrollment tertiary-center visit.

### Data collection

Quantitative data were collected at each follow-up visit through a structured visit fidelity checklist completed by the attending gynecologist and a patient-completed cost and time log documenting round-trip travel time, transport expenses (RWF), and workdays lost. Patient baseline travel time and cost data were collected retrospectively at enrollment to support pre–post comparisons. Additionally, the validated AIM, IAM, and FIM were administered verbally by the interviewer during a CFIR-aligned, one-on-one semi-structured qualitative interview. Participants responded to each item using the original 5-point Likert response options, and scores were calculated according to standard instrument procedures. Because AIM, IAM, and FIM were interviewer-administered rather than self-completed, social-desirability and mode-effect biases are possible and were considered in interpretation.

### Sample size rationale

No formal power calculation was performed. A target of 12 participants (∼3 per site across 4 district hospitals) was selected to identify major implementation barriers, estimate completion proportions, and identify the major recurring contextual determinants across sites.^18^ All quantitative findings are preliminary and hypothesis-generating and are designed to optimize the intervention and generate parameters for a future adequately powered implementation-effectiveness trial.

### Data analysis

Quantitative data were summarized using descriptive statistics. Proportions (visit completion, fidelity rates) were reported with exact Clopper–Pearson 95% confidence intervals (CIs). Qualitative interviews were analyzed using the framework method, with transcripts independently coded by 2 analysts (V.K. and F.R.) against a CFIR-domain coding framework (Inner Setting, Outer Setting, Characteristics of the Intervention, Implementation Process, Individuals). Discrepancies were resolved through discussion until consensus was reached. A realist-informed logic was layered on top of the framework method to explain inter-site variability: Coded data were synthesized into Context–Mechanism–Outcome configurations to explain observed implementation patterns and inter-site variability. Quantitative and qualitative findings were prospectively triangulated: quantitative outcomes defined the explanatory questions addressed in qualitative analysis.

### Ethics

Ethical approval was obtained from the Rwanda National Ethics Committee (143/RNEC/2023). Written informed consent was obtained from all participants prior to enrollment.

## Results

### Participant characteristics

Twelve cervical cancer survivors were enrolled across 4 district hospitals. The median age was 69 years (interquartile range [IQR] 54-72). Most had locally advanced disease: FIGO stage III (*n* = 6, 50.0%), stage II (*n* = 5, 41.7%), and stage IVA (*n* = 1, 8.3%). All participants had completed primary concurrent cisplatin-based chemoradiotherapy. Ten participants (83.3%) were HIV-seronegative. Participant characteristics are summarized in Table 1.

**Table 1:** Participant characteristics (*N* = 12).

Characteristic	*n* (%) or median (IQR)
**Age, years—median (IQR)**	69 (54-72)
**FIGO stage at enrollment**	
** Stage II**	5 (41.7)
** Stage III**	6 (50.0)
** Stage IVA**	1 (8.3)
**HIV status**	
** HIV-negative**	10 (83.3)
** HIV-positive [on antiretroviral therapy(ART)]**	2 (16.7)
**Highest educational attainment**	
** Primary school**	6 (50.0)
** Secondary school**	1 (8.3)
** University**	1 (8.3)
** Not educated**	4 (33.3)

Abbreviation: IQR, interquartile range.

### Implementation outcomes

#### Feasibility

Of 60 scheduled quarterly visits, 51 (85.0%; 95% CI, 73.4-92.9) were completed within the pre-specified 14-day window, surpassing the primary feasibility threshold of ≥80%. Site-level completion rates ranged from 66.7% to 93.8% (the study was not powered for inter-site comparison) (Table 2). Reasons were documented for all 9 missed visits and comprised provider unavailability due to competing clinical duties (*n* = 2), patient transport barriers (*n* = 3), and participant illness (*n* = 4). Laboratory and imaging investigations requested during follow-up visits were completed in 100% of cases, noting the small absolute number of tests (18/18 imaging; 5/5 lab tests), exceeding the pre-specified feasibility threshold of 90%. These small denominators should be interpreted with caution.

**Table 2:** Follow-up visit completion by site.

Site	Scheduled visits (*n*)	Completed visits (*n*)	Completion rate (%)	Exact 95% confidence interval (%)
**Ruhengeri and Burera DH—Northern Province (*n* = 6)** ^a^	32	30	93.8	79.2-99.2
**Nyagatare DH—Eastern Province (*n* = 3)**	12	8	66.7	34.9-90.1
**Kirehe DH—Eastern Province (*n* = 2)**	10	8	80.0	44.4-97.5
**Munini DH—Southern Province (*n* = 1)**	6	5	83.3	35.9-99.6
**Total (*N* = 12)**	60	51	85.0	73.4-92.9

a Ruhengeri and Burera DH represents a combined Northern Province catchment.

#### Acceptability and appropriateness

Clinicians rated the model as highly acceptable (AIM mean 4.5, SD 0.6), appropriate (IAM mean 4.3, SD 0.5), and feasible to implement (FIM mean 4.3, SD 0.5), all substantially exceeding the prespecified threshold of ≥4.0. Patients also rated the model as highly acceptable (AIM mean 4.4, SD 0.5) and appropriate (IAM mean 4.2, SD 0.4). Observed score ranges were narrow (clinician AIM 4.0-5.0, IAM 4.0-5.0, FIM 4.0-5.0; patient AIM 4.0-5.0, IAM 4.0-5.0). Critically, 100% of both clinician and patient respondents scored ≥4 on all applicable measures, with no dissatisfaction outliers across any of the 4 sites. Specific scores are presented in Table 3.

**Table 3:** Acceptability (AIM), appropriateness (IAM), and feasibility (FIM) scores by respondent group.

Respondent group	AIM mean (SD)	IAM mean (SD)	FIM mean (SD)	Threshold met?
**Clinicians (*n* = 4)**	4.5 (0.6)	4.3 (0.5)	4.3 (0.5)	MET (100% ≥4)
**Patients (*n* = 12)**	4.4 (0.5)	4.2 (0.4)	N/A	MET (100% ≥4)

Abbreviations: AIM, acceptability of intervention measure; FIM, feasibility of intervention measure—assessed among clinicians only; IAM, intervention appropriateness measure; N/A, not applicable; SD, standard deviation.

#### Fidelity

Of the 51 completed visits, charts for 46 (90.2%) were available for structured fidelity audit; 5 charts were missing during the time of analysis. Among these 46 visits reviewed for fidelity, the median visit-level fidelity was 64.2% (IQR 58.9%-67.4%), which was below the prespecified high-fidelity threshold of ≥85% (Table 4). Only 2 of 46 visits (4.3%) met this threshold. Site-level mean fidelity ranged from 53.9% to 69.7%, with no site achieving ≥85% fidelity in more than 10% of visits (Table 4). A key distinction was that baseline implementation fidelity was complete, with 100% of participants receiving an ASCO Survivorship Care Plan, a structured referral note sent from the cancer center for all participants, and all gynecologists and research assistants completing pre-study training.

**Table 4:** Visit-level protocol fidelity by site.

Site	Reviewed visits (*n*)	Mean fidelity (%)	Visits ≥85% Fidelity (%)	Baseline Fidelitya (%)
**Ruhengeri DH—Northern Province (*n* = 5)**	24	63.8	4.2% (1/24)	100
**Nyagatare DH—Eastern Province (*n* = 3)**	10	70.2	10.0% (1/10)	100
**Kirehe DH—Eastern Province (*n* = 2)**	8	64.5	0.0% (0/8)	100
**Munini DH—Southern Province (*n* = 1)** ^a^	4	53.9	0.0% (0/4)	100
**Overall**	46	Median 64.2% (IQR 58.9%-67.4%)	4.3% (2/46 visits)	100% all sites

a Only 1 participant enrolled at this site; estimates from this site should be interpreted with caution. Abbreviation: IQR, interquartile range.

#### Safety

No serious adverse events attributable to district-level follow-up were identified under passive surveillance via clinical-record and referral review; prospective adverse-event capture was not in place, and the small sample limits inference about safety. Four referrals occurred during the study period (2 for symptomatic post-radiation cystitis, 1 for a suspicious pelvic mass, and 1 for severe lymphedema). All patients requiring further evaluation were referred via the predefined pathway, and all documented referrals were completed within 14 days of referral; none exceeded the prespecified 7-day escalation window for red-flag triggers.

#### Patient time and financial burden

Follow-up at district-hospital reduced median round-trip travel time by 75% (from 8.0 to 2.0 hours per visit) and median transportation cost by 71% (from 12 000 to 3500 RWF per visit). Over 12 months with 4 scheduled quarterly visits, this represents an estimated per-participant saving of approximately 34 000 RWF (∼USD 23) in transportation costs (USD conversion based on the National Bank of Rwanda mid-market rate as of March 1, 2026, 1 USD ≈ 1460 RWF). Because tertiary-center values were retrospectively self-reported at enrollment, the pre–post comparison is approximate rather than fully prospective. The median workdays lost per visit were 1.0 day for both visit types, although district-hospital visits eliminated the overnight stays and the multi-leg journeys required for tertiary-center attendance (Table 5).

**Table 5:** Patient travel time and financial burden: district-hospital versus tertiary-center follow-up.

Metric	Tertiary center median (IQR)	District hospital median (IQR)	Reduction (%)	Median difference (IQR)
**Round-trip travel time (hours)**	8.0 (7.5-10.0)	2.0 (1.0-2.0)	75	6.0 (5.0-8.0)
**Out-of-pocket transport cost (RWF)**	12 000 (11 500-12 000)	3500 (2500-6000)	71	8500 (5500-9500)
**Workdays lost per visit (days)**	1.0 (1.0-1.0)	1.0 (1.0-1.0)	0	0.0 (0.0-0.0)
**Estimated per-participant annual transport saving (RWF)**	—	—	∼34 000 RWF (∼USD 23)	—

Abbreviations: IQR, interquartile range RWF, Rwandan francs.

#### Summary against prespecified implementation criteria

A structured summary of all prespecified implementation outcomes against benchmarks is in Table 6. Three principal conclusions emerge: (1) the on-time visit completion threshold (≥80%) was met (85.0%; 95% CI, 73.4-92.9); (2) acceptability and appropriateness criteria were exceeded, with 100% of respondents achieving ≥4 on all AIM/IAM/FIM measures; and (3) visit-level protocol fidelity was substantially below the high-fidelity benchmark (median 64.3%; 4.3% of visits achieving ≥85%), representing the primary implementation gap for targeted intervention before scale-up.

**Table 6:** Summary of implementation outcomes against prespecified criteria.

Implementation outcome	Prespecified threshold	Observed result	Criterion met?
**Visit completion**	≥80%	85.0% (95% CI, 73.4-92.9) 51/60 visits attended	MET
**Clinically indicated test completion**	≥90%	100% of tests requested were done	MET
**Acceptability—patients (AIM)**	≥4.0; 100% ≥4	AIM 4.4 (SD 0.5); 100% ≥4	MET
**Acceptability—clinicians (AIM)**	≥4.0; 100% ≥4	AIM 4.5 (SD 0.6); 100% ≥4	MET
**Appropriateness—clinicians (IAM)**	≥4.0; 100% ≥4	IAM 4.3 (SD 0.5); 100% ≥4	MET
**Clinician feasibility (FIM)**	≥4.0; 100% ≥4	FIM 4.3 (SD 0.5); 100% ≥4	MET
**Visit-level protocol fidelity**	≥85% per visit	Median 64.2% (IQR 58.9%-67.4%) 4.3% visits ≥85%	NOT MET
**Safety (no serious AEs attributable to decentralization)**	Zero serious AEs	No serious AEs identified (passive surveillance only)c	MET^a^
**Referral/escalation when clinically indicated**	100% timely	Completed in all documented cases	MET

Abbreviations: AIM, acceptability of intervention measure; AE, adverse event; CI, confidence interval; FIM, feasibility of intervention measure; IAM, intervention appropriateness measure; IQR, interquartile range; SD, standard deviation.

a Safety criterion met under passive surveillance only; prospective structured safety monitoring is required before scale-up.

#### Qualitative findings

CFIR-informed analysis of interviews with 12 patients and 4 gynecologists identified 4 thematic domains.

1. Inner Setting: District hospitals were seen as having adequate infrastructure; gynecologists expressed confidence in their clinical competence. However, occasional medication stock-outs and competing workload demands—particularly at busier sites where shortened visit duration contributed to documentation omissions—provided the mechanistic explanation for the acceptability–fidelity dissociation.
2. Outer Setting: Rwanda’s Mission 2027 elimination goal was mentioned as a legitimizing force enabling clinician engagement. The financial burden on patients emerged as the most significant driver of the model’s value: “I get there quickly and return home quickly” [PT1]; “Here I pay five hundred francs but going to Kigali I pay around ten thousand” [PT7].
3. Characteristics of the Intervention: The ASCO Survivorship Care Plan was valued as a transition document; structured checklists converted abstract protocol expectations into actionable visit tasks [Gyn4]; and telephone follow-ups by research assistants were identified as critical engagement mechanisms.
4. Implementation Process: Pre-study training was necessary, but insufficient; clinicians still reported residual gaps in late-effect management, reinforcing the need for refresher sessions. The referral pathway was described as functional and trust-building by both clinicians (“If I can’t manage it, there is a clear path” [Gyn2]) and patients (“If things became serious, he would refer me again” [PT1]). Structured reminders were essential to ensure adherence to visits (“They used to call me and remind me it was time to see the doctor” [PT3]). Full theme mapping is provided in Table 7.

**Table 7.** CFIR-informed qualitative themes and representative quotes.

Theme	CFIR domain/construct	Representative patient quote	Representative clinician quote
**Acceptability and trust in the model**	Intervention characteristics—relative advantage; process—engaging	“I trusted the district hospital doctor… if things became serious, he would refer me again.” [PT1]	“When follow-up is only at the referral center, patients often fail to come because it is far.” [Gyn3]
**Reduced financial and logistical burden**	Outer setting—patient needs and resources	“I pay five hundred francs here but going to Kigali I pay around ten thousand.” [PT7]	“Most of our patients come from poor households; traveling to Kigali is very difficult.” [Gyn1]
**Provider competence and quality of care**	Characteristics of individuals—knowledge and beliefs; intervention design	“He examined me thoroughly and explained everything clearly.” [PT10]	“The checklist makes things easier because what we are supposed to check is clear.” [Gyn4]
**Health system readiness and resource constraints**	Inner setting—available resources; workload and competing demands	“The care here is the same as what I received at the cancer center.” [PT4]	“We use the same tools for other patients; there is nothing unusual—but stock-outs can delay care.” [Gyn1]
**Continuity, coordination and reminder systems**	Process—executing; networks and communication	“They used to call me and remind me it was time to see the doctor.” [PT3]	“When referral is needed, we call the receiving doctor and organize the appointment.” [Gyn1]

Abbreviations: CFIR, consolidated framework for implementation research; Gyn, gynecologist participant; PT, patient participant (coded).

## Discussion

This study aimed to evaluate whether gynecologist-led cervical cancer survivorship follow-up at district hospitals in Rwanda is feasible, acceptable, and safe. Five major findings were identified: First, on time visit completion (within 14 days of the planned date) met the prespecified feasibility threshold (85.0% vs. ≥80%). Second, the model was consistently and highly acceptable to both patients and clinicians. Third, visit-level protocol fidelity was considerably below the high-fidelity threshold despite completion of baseline implementation requirements; this dissociation between high acceptability and low visit-level fidelity is the principal conceptual contribution of the study. Fourth, decentralized follow-up reduced median travel time and transport cost, with expected downstream benefits for adherence, continuity of care, and equity for older, rural, and lower-income survivors and finally, under passive surveillance and a small sample, no serious adverse events attributable to district-level follow-up were identified and all documented referrals were completed; prospective adverse-event capture was not in place and a formal safety claim cannot be made.

To our knowledge, this is among the first prospective, multi-site studies to evaluate implementation outcomes of decentralized cervical cancer survivorship care at district hospitals in an LMIC. The cohort’s age and stage distribution are broadly consistent with the Rwandan cervical-cancer population presenting.^4^ A recent scoping review of cancer survivorship in Africa (2011-2024) found that post-treatment care remains primarily focused on psychosocial outcomes, with limited attention to care coordination or formal implementation outcome assessment.^26^ Our study directly addresses this gap using validated instruments within a theoretically grounded framework.

The AIM/IAM/FIM scores support preliminary acceptability of both the instruments and the intervention in this context and align with psychometric validation studies^25^ and cross-cultural adaptation data,^27^ supporting the potential applicability of these measures in LMIC settings. The key finding that high acceptability coexisted with low fidelity provides empirical support for the conceptual distinction between these outcomes emphasized in Proctor’s taxonomy,^17^ a pattern described theoretically but rarely demonstrated with prospective data in LMIC oncology.^28^ The CFIR-informed qualitative findings, particularly the role of workload constraints in driving documentation omissions, align with Schmitt et al.’s systematic review, identifying inner-setting resource pressures as the most common barriers to fidelity.^19^ They also mirror Kenya’s cancer decentralization experience, where national policy alignment facilitated adoption at regional centers.^29^

The 75% reduction in travel time and 71% reduction in transport cost are consistent with the substantial access benefits expected from cancer care decentralization in sub-Saharan Africa. Previously our team^14^ demonstrated significant financial toxicity among Rwandan cancer patients, with 44% reporting to have sold property to fund treatment costs, while only 13% were able to access a cancer center within an hour.^13^ Our findings suggest that geographic proximity is crucial for follow-up adherence, continuity of care and equity aligning with evidence from South Africa, where decentralization substantially reduced the financial burden of cancer care.^30^ The fidelity gap observed (median 64.3%) is not uncommon, Stockton et al. reported intervention fidelity ranging from 54% to 95% across NCD clinics in Malawi, with audit-and-feedback strategies achieving 82%-91% fidelity compared to 61%-93% for standard approaches,^31^ providing direct evidence that structured feedback can improve protocol adherence in LMIC health systems.

### Strengths and weaknesses

This study has several limitations. First, the sample size was small (12 participants). Second, site-level numbers were too limited for formal inter-site comparisons, and we acknowledge purposeful sampling may limit generalizability to lower-capacity district hospitals without on-site gynecologists. Third, fidelity assessment measured completion of required elements but not clinical quality; omitted documentation may also have concealed omitted clinical actions, with direct patient-safety implications. Fourth, AIM, IAM, and FIM were interviewer-administered, raising the possibility of social-desirability and mode-effect biases, which could help explain the uniformly high scores. Fifth, the intervention included quarterly research-assistant telephone reminders, which were a research-support component and may not be available under routine implementation; this support may have improved on-time attendance, and care should be taken not to over-attribute feasibility to the model alone. Additionally, no clear adherence or acceptability gradient by educational attainment was observed; however, the small sample precludes formal analysis. Qualitative interviews suggested phone reminders mitigated literacy barriers. Sixth, safety surveillance was passive, relying on clinical-record and referral review rather than prospective adverse-event capture, so a formal safety claim cannot be supported by these data. Seventh, retrospectively self-reported pre-enrollment travel data may have introduced recall bias. Despite these limitations, the study is strengthened by its prospective multi-site LMIC design, a priori outcome definitions based on Proctor’s taxonomy,^17^ use of validated implementation measures,^25^^,^^27^ dual-coded CFIR-based qualitative analysis,^19^^,^^20^ quantitative-qualitative triangulation, and inclusion of 4 geographically diverse sites.

### Implications for practice and future research

The main contribution of this study is demonstrating that district-hospital based survivorship care follow-up is feasible in terms of on-time visit completion and highly valued by patients and clinicians in this LMIC setting, while achieving consistent visit-level fidelity will require additional intervention before scale-up. Omitted documentation of pelvic findings, investigations, and referral decisions should be understood not only as an implementation gap but also as a potential quality-of-care and recurrence-surveillance risk. Prior to this study, there was no prospective evidence showing whether such care is feasible or safe in sub-Saharan Africa.^26^ Three practice implications emerge. First, the acceptability–fidelity dissociation shows that acceptability cannot be used as a substitute for quality; prospective fidelity monitoring must be integrated from the beginning. The most omitted elements-documentation of examination findings, investigations, and referral decisions are among the most crucial for monitoring recurrence, and their omission poses a patient safety concern. Second, reducing patient burden is fundamentally important; any scale-up that dilutes the advantage of proximity would weaken the model’s effectiveness. Third, passive safety surveillance alone is not enough for scale-up; a minimum viable system must include standardized documentation of red-flags, mandatory referral records, and prospective linkage with tertiary records.

A definitive implementation-effectiveness trial should incorporate a fidelity-strengthening package with simplified visit checklists, regular audit and feedback,^31^^,^^32^ and peer learning sessions; prospective structured safety monitoring; formal cost-effectiveness analysis; sufficiently powered patient-reported outcome assessment; and extended follow-up to assess recurrence detection. Rwanda’s Mission 2027 strategy^5^ provides a natural policy framework, and following intervention refinement (fidelity-strengthening package and structured prospective safety monitoring), the convergence of workforce availability, patient demand, and national commitment creates favorable conditions for a subsequent stepped-wedge trial across additional district hospitals.^33^^,^^34^

## Conclusions

Gynecologist-led cervical cancer survivorship follow-up at district hospitals in Rwanda was acceptable to patients and clinicians and substantially reduced travel time and transport costs compared with tertiary-center attendance; on-time visit completion met the prespecified threshold, while visit-level protocol fidelity remained low. The model is promising but requires a fidelity-strengthening package and structured prospective safety monitoring before broader implementation. A definitive implementation-effectiveness trial incorporating a fidelity-strengthening package, prospective safety monitoring, and formal cost-effectiveness analysis is warranted and should be prioritized within Rwanda’s Mission 2027 cancer survivorship agenda.

## Data Availability

Data generated during this study is available from the corresponding author on reasonable request.
